# Identification of ε-Poly-L-lysine as an Antimicrobial Product from an *Epichloë* Endophyte and Isolation of Fungal ε-PL Synthetase Gene

**DOI:** 10.3390/molecules25051032

**Published:** 2020-02-25

**Authors:** Enkhee Purev, Tatsuhiko Kondo, Daigo Takemoto, Jennifer T. Niones, Makoto Ojika

**Affiliations:** 1 Department of Applied Biosciences, Graduate School of Bioagricultural Sciences, Nagoya University, Chikusa-ku, Nagoya 464-8601, Japan; enkheepurev@gmail.com (E.P.); kontatsu@agr.nagoya-u.ac.jp (T.K.); 2 Department of Plant Production Sciences, Graduate School of Bioagricultural Sciences, Nagoya University, Chikusa-ku, Nagoya 464-8601, Japan; dtakemo@agr.nagoya-u.ac.jp (D.T.); jenniferniones@gmail.com (J.T.N.); 3 Philippine Rice Research Institute, Science City of Munoz, Nueva Ecija 3119, Philippines

**Keywords:** ε-polylysine, *Epichloë*, endophyte, fungicide, transcription factor

## Abstract

The endophytic fungus *Epichloë festucae* is known to produce bioactive metabolites, which consequently protect the host plants from biotic and abiotic stresses. We previously found that the overexpression of *vibA* (a gene for transcription factor) in *E. festucae* strain E437 resulted in the secretion of an unknown fungicide. In the present study, the active substance was purified and chemically identified as ε-poly-L-lysine (ε-PL), which consisted of 28–34 lysine units. The productivity was 3.7-fold compared with that of the wild type strain E437. The isolated ε-PL showed inhibitory activity against the spore germination of the plant pathogens *Drechslera erythrospila*, *Botrytis cinerea*, and *Phytophthora infestans* at 1–10 μg/mL. We also isolated the fungal gene “*epls*” encoding ε-PL synthetase Epls. Overexpression of *epls* in the wild type strain E437 resulted in the enhanced production of ε-PL by 6.7-fold. Interestingly, overexpression of *epls* in the different strain *E. festucae* Fl1 resulted in the production of shorter ε-PL with 8–20 lysine, which exhibited a comparable antifungal activity to the longer one. The results demonstrate the first example of ε-PL synthetase gene from the eukaryotic genomes and suggest the potential of enhanced expression of *vibA* or/and *epls* genes in the *Epichloë* endophyte for constructing pest-tolerant plants.

## 1. Introduction

Endophytes are a heterotrophic group of microorganisms including bacteria, actinomycetes and fungi that colonize in plant tissues. Endophytic fungi exhibit a considerable reservoir of unexplored microorganisms capable of producing novel metabolites [1]. Among them, the genus *Epichloë* belongs to the fourth dominant class Sordariomycetes in Ascomycete endophytic fungi [2]. Their mutual interaction benefits the host plants by protecting them from insects [3,4] and diseases [5,6] and by increasing their tolerance against environmental stresses such as drought [7]. An enhanced host plant tolerance against pests is the most significant advantage for the plants because of bioactive secondary metabolites produced by endophytic fungi [8]. To date, a variety of bioactive secondary metabolites have been isolated and characterized from the *Epichloë* endophytes. They have been known as abundant sources of insecticide alkaloids such as loline and peramine [9]. Indole-3-acetic acid, indole-3-ethanol, methylindole-3-carboxylate, indole-3-carbox-aldehyde, *N*,*N′*-diacetamide, and cyclonerodiol were isolated from cultures of *E. festucae* isolates as antifungal agents against grass pathogens [10]. A cyclic peptide, epichlicin, produced by *E. typhina* inhibits the spore germination of *Clasdosporium phlei* [11]. Seventeen antifungal metabolites, including a new product, 3-(2′-(4″-hydroxyphenyl)acetoxy)-2*S*-methylpropanioc acid, were isolated from *E. bromicola* [12]. We previously identified *E. festucae* strain E437 as a producer of antifungal substance against several grass pathogens such as *Drechslera erythrospila*, *Colletotrichum graminicola*, and *Bipolaris sorokiniana* [13]. We have isolated a mutant that had lost antifungal activity by random mutagenesis, and a transcription factor VibA was identified as an essential factor for the production of the antifungal activity [14]. Fungal VibA homologs (also known as VIB-1) are NDT80/PhoG-type transcription factors that regulate a wide range of genes involved in the vegetative incompatibility, conidiation, formation of aerial hyphae, development of female reproductive structure, and production of extracellular protease [15,16,17]. Overexpression of *VibA* gene promoted the production of an unknown antifungal substance against plant pathogens [14]; however, identification of the antifungal product was remained to be solved. The present study highlights the purification, chemical characterization, and biosynthesis of the unknown antifungal substance produced by the *Epichloë* endophyte.

## 2. Results

### 2.1. Chemical Identification of an Antifungal Substance Produced by a VibA-Overexpressed Strain of E. festucae E437

The *vibA* gene-overexpressing transformant P*tef::VibA* (E437) derived from *E. festucae* strain E437 (wild type) was cultured in a liquid PD medium. A culture filtrate was found to inhibit conidial germination of the plant pathogen *D. erythrospila*. It was previously shown that the activity was 10 times as high as that of the original E437 strain [14]. The active filtrates were subjected to three-steps purification: (1) cation exchange resin (Amberlite-IRC 76), (2) reversed-phase HPLC (ODS column), and (3) normal-phase HPLC (HILIC column), affording an active substance in the yield of 38.6 mg/L. The chemical structure of the active substance was analyzed by NMR spectroscopy in D_2_O (Supplementary Materials, Figures S1–S5). A heteronuclear multiple quantum correlation (HSQC) spectrum suggested the presence of one CH and four CH_2_ groups. One quaternary carbon atom observed at 169.6 ppm suggested the presence of one carboxyl group. Double quantum-filtered correlation spectroscopy (DQF-COSY) revealed the important partial structure of >CH-CH_2_-CH_2_-CH_2_-CH_2_-. A hetero-nuclear multiple-bond connectivity (HMBC) experiment was next performed to connect the partial structure and the carboxyl group. The HMBC correlations were found between C=O and the protons of Hα, Hβ, and Hε (Table 1), indicating that the structure was a cyclic form of lysine (α-amino-ε-caprolactam) or ε-polylysine (ε-PL).

**Figure 1:**
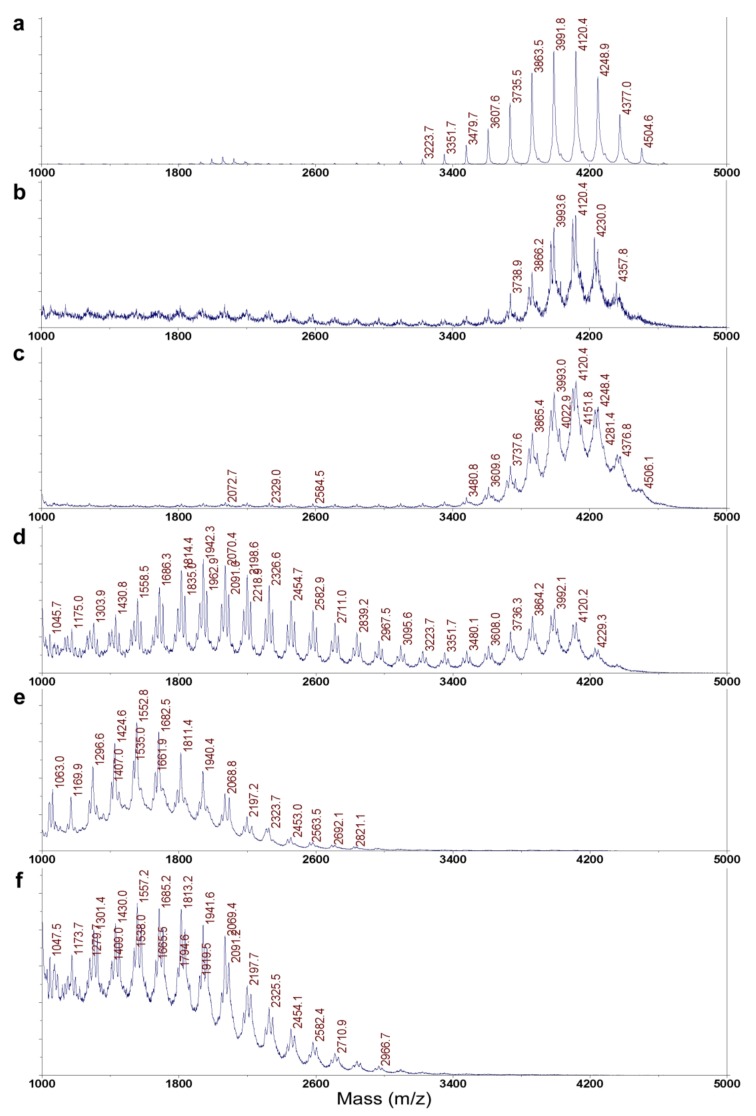
Matrix-Assisted Laser Desorption Ionization-Time-of-Flight Mass Spectroscopy (MALDI-TOF MS) of ε-PL. (**a**) Standard ε-PL; (**b**) ε-PLs from *E. festucae* strain E437 (wild type); and (**c**–**f**) ε-PLs from the transformants P*tef::VibA* (E437), P*tef::Epls* (E437), P*tef::VibA* (Fl1), and P*tef::Epls* (Fl1), respectively.

The structure of the active substance was finally determined as ε-PL by Matrix-Assisted Laser Desorption Ionization-Time-of-Flight Mass Spectroscopy (MALDI-TOF MS), and the degree of polymerization was found to be 28–34, which was the same as that of standard ε-PL (Figure 1a,c). The ^1^H NMR was superimposable to that of a standard e-PL (bacterial origin, triflouroacetic acid (TFA) salt) (Supplementary Materials, Figure S6). The absolute configuration of the lysine unit was determined as L by the advanced Marfey’s method [18] using L- and D-1-fluoro-2,4-dinitrophenyl-5-leucinamides (FDLA) (Supplementary Materials, Figure S7). Therefore, we concluded that the antifungal substance produced by P*tef::Epls* (E437) strain was ε-poly-L-lysine (ε-PL).

### 2.2. Identification of ε-PL Biosynthetic Gene Epls from E. festucae 

**Figure 2:**
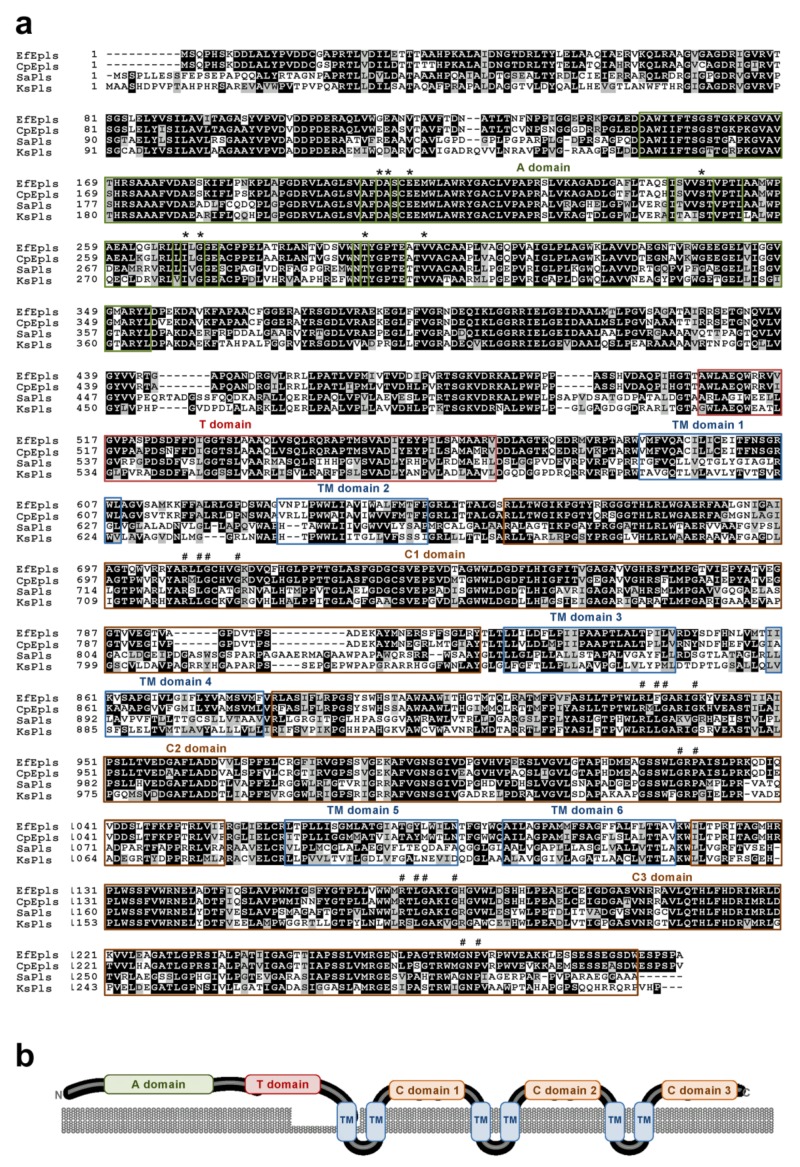
Structure of ε-poly-_L_-lysine synthetase of *E. festucae*. (**a**) Alignment of the deduced amino acid sequences of fungal and bacterial ε-poly-L-lysine synthetases (Epls and Pls, respectively). Nonribosomal peptide synthetases adenylation (A) and thiolation (T) domains; transmembrane (TM) domains; and C-terminal tandem C1, C2, and C3 domains are indicated by green, red, blue, and orange, respectively. Amino acid residues for the substrate-binding pocket in A domain [21] are indicated by asterisks, and conserved amino acids in tandem C domains for lysine polymerization [23] are indicated by hash marks. Ef, *Epichloë festucae*; Cp, *Claviceps purpurea*; Sa, *Streptomyces albulus*; and Ks, *Kitasatospora setae*. (**b**) Predicted architecture of *E. festucae* Epls.

**Figure 3:**
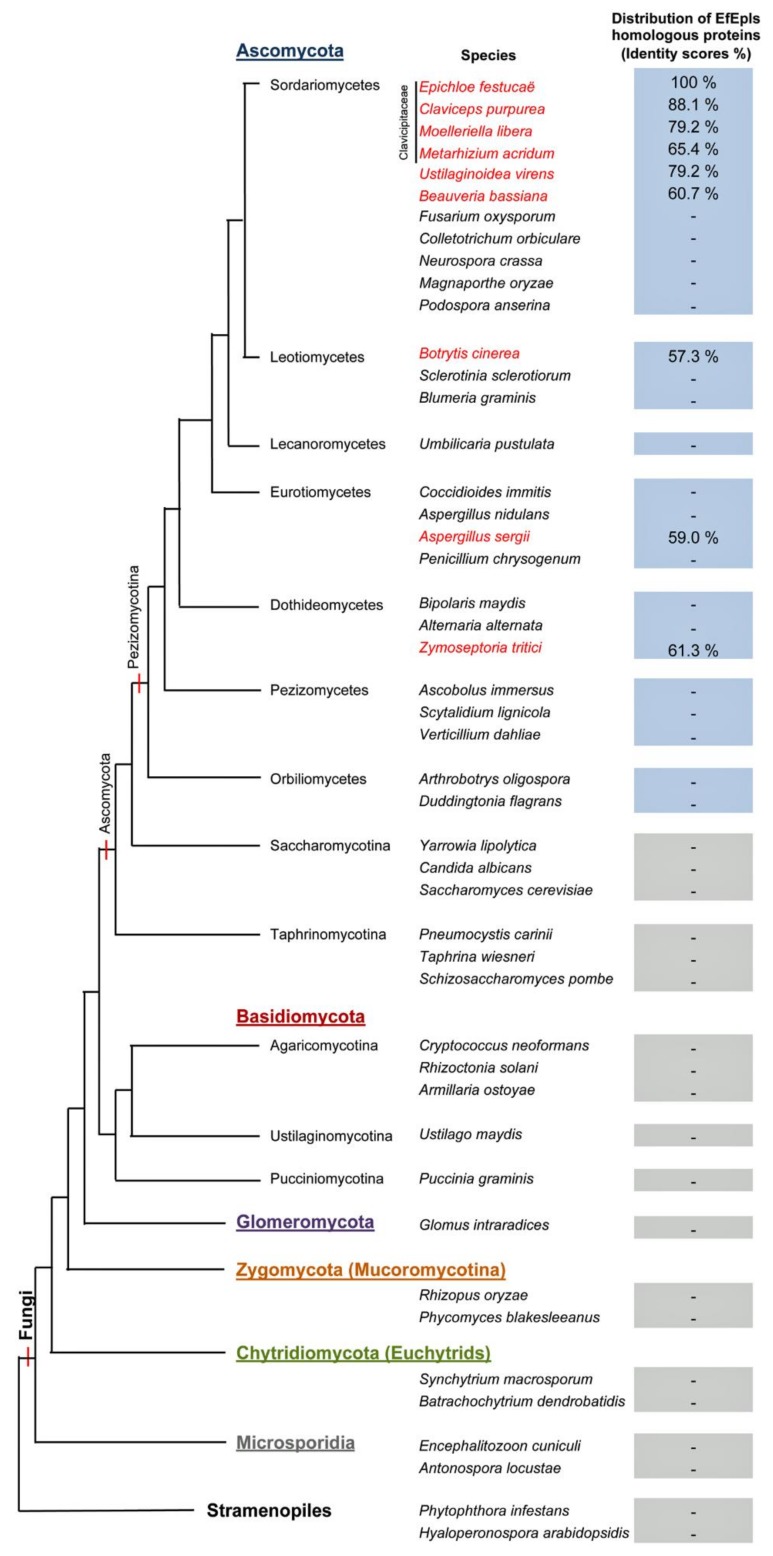
Distribution of Epls homologues in the fungi kingdom. Percentage identities of deduced amino acid sequence with *E. festucae* Epls were calculated with MacVector program (ver. 15) with the default setting.

The genes for ε-PL biosynthetic enzyme Pls (ε-poly-L-lysine synthetase) have been identified from bacterial species, such as *Streptomyces albulus* and *Kitasatospora setae.* Bacterial Pls is a nonribosomal peptide synthetase that has N-terminal lysine-binding domain and C-terminal tandem domains for polymerization of lysine [19,20]. However, the corresponding genes have not been isolated from fungi. Using *S. albulus* Pls as the query of a blastp search, EfM3.024590 was identified from the genome sequence of the endophytic fungus *E. festucae* as a candidate gene for Pls. Although identity of amino acid sequences between *S. albulus* Pls and *E. festucae* EfM3.024590 is only 51%, they have a similar domain architecture, including N-terminal adenylation (A) and thiolation (T) domains for nonribosomal peptide synthetases and C-terminal tandem C1, C2, and C3 domains, which are unique to Pls (Figure 2). The substrate-binding pocket of adenylation domains [21] is conserved among bacterial Pls, EfM3.024590, and the lysine-binding pocket in the first A domain of the *Bacillus licheniformis* bacitracin synthetase 2 (Figure 2 and Supplementary Materials, Figure S8). Therefore, EfM3.024590 was designated as Epls for the *E. festucae* ε-PL synthetase. We here propose the name “Epls” for the fungal Pls, as the name Pls is widely used for a component of fungal enzymes for the production of reactive oxygen species (e.g., [22]). Homologues of Epls are found in a few Ascomycota fungi and well conserved, especially in the family Clavicipitaceae, including some plant- and entomo-pathogenic fungi (Figure 3).

### 2.3. Overexpression of Epls Gene in E. festucae Strains

*E. festucae* strain E437 produces an antifungal substance, while a culture filtrate of strain Fl1 shows no detectable antifungal activity [13]. Overexpression of *vibA* in E437, P*tef::VibA* (E437) strain, and that in Fl1, P*tef::VibA* (Fl1) strain, showed the enhancement of the antifungal activity compared with their wild type strains [14]. Although this phenomenon suggested that the transcription factor gene *vibA* is involved in the biosynthesis of e-PL, the direct evidence of the participation of the *epls* gene in *E. festucae* was still unclear. 

To investigate the role of the *E. festucae epls* gene, *E. festucae* strains E437 and Fl1 were transformed with a vector for the overexpression of the *epls* gene under the control of the TEF (translation elongation factor) promoter [24]. ε-PL was purified from the obtained transformants P*tef::Epls* (E437) and P*tef::Epls* (Fl1), as well as the previously obtained P*tef::VibA* (Fl1), and analyzed by MALDI-TOF MS (Table 2). The production of ε-PL was enhanced by 6.7-fold in P*tef::Epls* (E437) and infinite in P*tef::Epls* (Fl1), as much as that of the wild type strains. On the other hand, the production of ε-PL was enhanced more than 3.7-fold in the P*tef::VibA* (E437) strain and infinitely in P*tef::VibA* (Fl1), more than their wild type strains. These results clearly demonstrate that not only *vibA* but also *epls* genes are involved in the biosynthesis of ε-PL in *E. festucae* and that their overexpression promotes ε-PL production. It is interesting to note that the degree of polymerization (DP) of ε-PL is drastically affected by host strains; (1) the Fl1 strains (two transformants) produced lower DP product (DP = 8–18 and 8–20; DP of largest population = 12), (2) the *epls*-overexpressing transformant P*tef::Epls* (E437) produced not only lower DP product but also a higher one (DP = 26–33; DP of largest population = 31), (3) the E437 strains (wild type and *vibA*-overexpressed transformant) exclusively produced higher DP products (DP = 28–34; DP of largest population = 32). 

### 2.4. Inhibitory Activity of ε-PL against Spore Germination of Fungal and Oomycete Pathogens 

**Figure 4:**
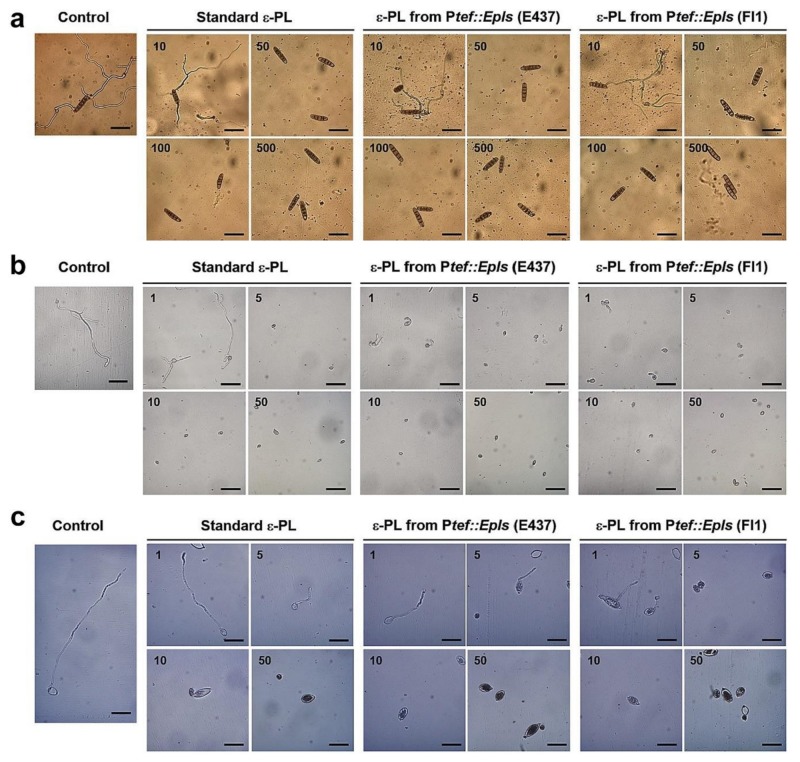
Inhibitory activity of ε-PL against spore germination of plant pathogens. (**a**) Conidia of *D. erythrospila*, (**b**) conidia of *B. cinerea*, and (**c**) zoosporangia of *P. infestans* were treated with triflouroacetic acid (TFA) salts of standard ε-PL and P*tef::Epls* (E437)- and P*tef::Epls* (Fl1)-derived ε-PL at the indicated concentrations (mg/mL) for 20 h. Bars = 50 µm.

**Figure 5:**
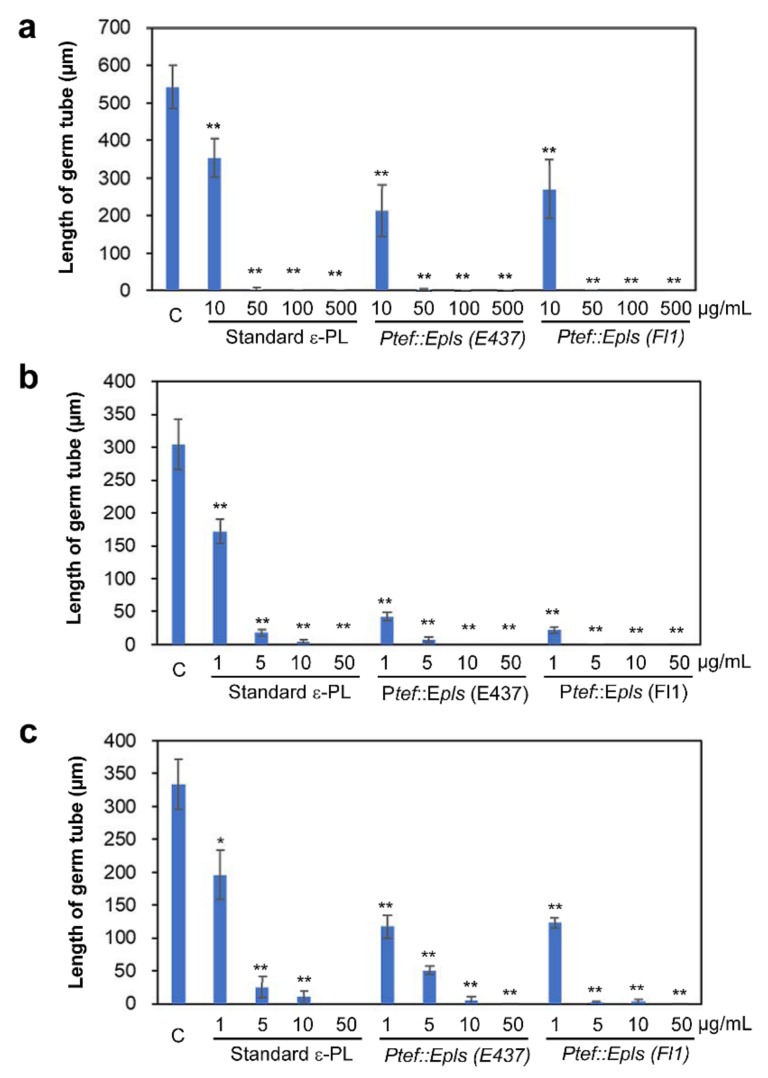
Quantitative analysis for inhibitory activity of ε-PL against spore germination and hyphal elongation of (**a**) *D. erythrospila*, (**b**) *B. cinerea*, and (**c**) *P. infestans*. Length of germinated hyphae was measured for the microscopic images shown in Figure 4. Data are means with standard errors (n = 6). Data marked with asterisks are significantly different from control (C), as assessed by two-tailed Student’s *t*-tests: * *p* < 0.05 and ** *p* < 0.01.

**Figure 6:**
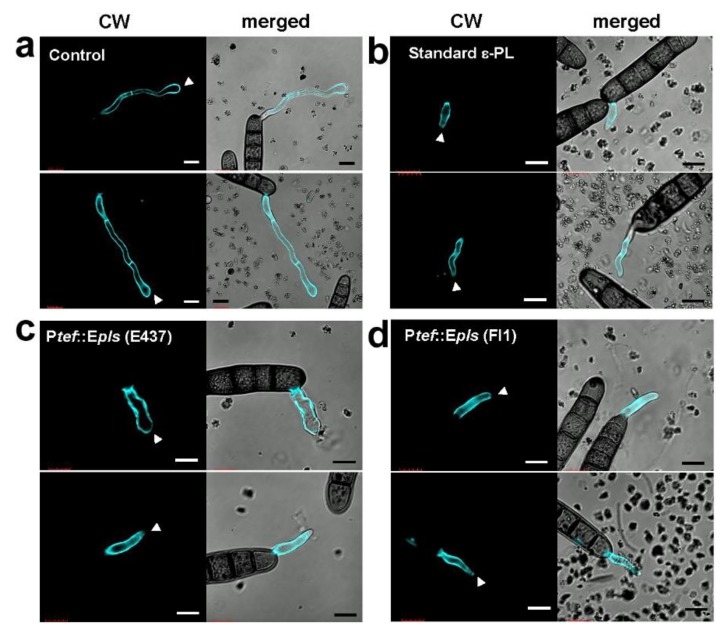
Morphology of germinated hyphae of *D. erythrospila* treated with ε-PL. Conidia of *D. erythrospila* were incubated with (**a**) water, (**b**) 50 µg/mL standard ε-PL, (**c**) 50 µg/mL ε-PL from P*tef::Epls* (E437), or (**d**) ε-PL from P*tef::Epls* (Fl1) for 20 h at 23 °C. Germinated hyphae were stained with Calcofluor White and observed under a confocal laser scanning microscope. Arrowheads indicate the tip of growing hyphae. Bars = 10 µm.

Three samples of ε-PL (as TFA salts), a standard bacterial ε-PL, P*tef::Epls* (E437)-derived ε-PL containing long chain one, and P*tef::Epls* (Fl1)-derived ε-PL containing only short chain one, were evaluated for the inhibition of spore germination and hyphal growth of fungal and oomycete plant pathogens: grass pathogen *D. erythrospila*, gray mold *B. cinerea*, and potato late blight pathogen *P. infestans*. The conidia or zoosporangia of pathogens were treated with ε-PL for 20 h, and the length of germinated hyphae was measured (Figure 4). The spore germination of *D. erythrospila* and the other two pathogens was almost suppressed by three ε-PL samples at 50 and 5 μg/mL, respectively (Figure 5). *B. cinerea* was most effectively inhibited by ε-PL (Figure 5b). The ε-PL with only short chain lengths isolated from P*tef::Epls* (Fl1) indicated a little higher activity than that containing long chain ones from P*tef::Epls* (E437). In order to investigate the effect of ε-PL in the growth of pathogens, germinating hyphae of *D. erythrospila* were stained with Calcofluor White reagent, which is a fluorescent dye that binds to the polysaccharide polymers on fungal cell-wall chitin. For germinating hyphae of *D. erythrospila*, the fluorescent dye was accumulated at the hyphal tip, where the cell wall is actively synthesized (Figure 6a, arrowheads). On the other hand, *D. erythrospila* hyphae treated with 50 µg/mL of standard ε-PL and P*tef::Epls* (E437)- or P*tef::Epls* (Fl1)-derived e-PL showed decreased accumulation of polysaccharide polymers at hyphae tips (Figure 6b–d), suggesting that ε-PL inhibited the polarized production of polysaccharide for the cell wall at the hyphal tip of *D. erythrospila.*

### 2.5. Antifungal Activity of ε-PL

The observation of the conidial germination inhibition against *D. erythrospila*, *B. cinerea*, and *P. infestans* prompted us to evaluate antifungal activity of ε-PL by a paper disk diffusion method against eight plant pathogens: *D. erythrospila*, *P. capsici, C. orbiculare, F. oxysporum*, *B. cinerea, M. oryzae, A. alternata*, and *A. niger*. The results showed that both ε-PL samples produced by P*tef::Epls* (E437) with long and short chain lengths and P*tef::Epls* (Fl1) with a short one showed the antifungal and anti-oomycete activities against only *D. erythrospila* and *P. capsici* at 300 μg/disk, which was comparable to that of standard ε-PL (Supplementary Materials, Table S1 and Figure S9). There was no clear polymer-size dependency in the antimicrobial activity of ε-PL. 

## 3. Discussion 

Infection of the fungal endophyte *E. festucae* E437 to perennial ryegrass enhanced disease resistance of the host plant to the grass pathogen *D. erythrospila* [13], suggesting that this endophyte produces unknown bioactive metabolite(s), which is effective to suppress the infection of fungal pathogens. It was actually observed in co-culture experiments that the endophyte grown on an agar medium inhibits the growth of *D. erythrospila*. The transformant *Ptef::VibA* (E437) that possesses an overexpressed *vibA* gene showed stronger antifungal activity than the wild type strain against a wide range of plant pathogens [14]. These phenomena attracted our attention and prompted us to reveal the chemical nature of the antifungal compound and its production mechanism. In this study, by culturing the transformant followed by chromatographic purification and spectroscopic analysis, the antifungal principle was identified as ε-poly-L-lysine (ε-PL) consisting of 24–35 lysine units for the first time.

ε-PL has been known as an antibacterial polyamine secreted mainly by some bacteria in the Streptomycetaceae family [25] and a few members of the Bacillaceae family [26,27]. Production of ε-PL in fungi has also been reported for Clavicipitaceae fungi, including ergot fungi *Claviceps purpurea* [28] and *Epichloë* sp. MN-9 [29]. Bacterial ε-PL is biosynthesized by the transmembrane protein ε-poly-l-lysine synthetase (Pls), which features nonribosomal peptide synthetase with adenylation and thiolation domains, six transmembrane, and three tandem domains [19]. After an in silico analysis, a gene encoding the protein with the same domain architecture was identified from the genome of *E. festucae*, which was designated as *epls* (Figure 2). A phylogenetic analysis revealed that homologues of the *E. festucae epls* are found mainly in Clavicipitaceae species, being consistent with the above-mentioned fungal producers of e-PL (Figure 3) Overexpression of the *epls* gene in *E. festucae* strains E437 enhanced the production of ε-PL.

Moreover, strain Fl1, a nonproducer of ε-PL, also produced ε-PL by overexpression of *epls* (Table 2), indicating that Epls is the biosynthetic enzyme (or at least responsible) for the production of ε-PL in this symbiotic fungus. Since the overexpression of the *vibA* gene in *E. festucae* also enhanced the production of e-PL and VibA is known as a transcription factor with diverse functions in Ascomycota fungi [17,30,31], it is expected that VibA directly regulates the expression of *epls*. However, our preliminary investigations indicated that the overexpression of *vibA* did not enhance the transcription of *epls* (data not shown). 

Thus, the role of *E. festucae* VibA in the ε-PL production remains to be determined. Interestingly, while the wild-type E437 strain and the P*tef::VibA* (E437) transformant produced ε-PL with long chain lengths (DP = 28–34), others also produced that with short chain lengths (DP = 8–18). Especially, the transformants derived from the Fl1 strain, P*tef::VibA* (Fl1) and P*tef::Epls* (Fl1), produced exclusively short chain polymers (Figure 1 and Table 2). While most of the ε-PLs originated from *Streptomyces* species are known to possess long chains (DP = approximately 26–35), only limited numbers of microorganisms have been identified as short-chain ε-PL producers [32]. Hamano et al. (2014) suggested that the linker regions connecting with the transmembrane domain of Pls were responsible for the shortening ε-PL length in *Streptomyces* species [20]. It was also reported that supplementing culture media with glycerol and glucose shortened the ε-PL chain length [33]. Among the constructed transformants, P*tef::Epls* (E437) was the best producer of ε-PL (approximately 70 mg/L). Since ε-PL has attracted attention as an antimicrobial agent with a wide inhibitory spectrum against Gram-positive and negative bacteria, fungi, yeasts, and phages [25], several strategies to improve the production of ε-PL have been conducted; for example, modifying culture conditions (supplementation with glucose or other metabolic precursors, modifying pH, etc.) [27,34], inducing double antibiotic-resistant mutations [35] and genome shuffling [36]. The present study suggests that the gene overexpression strategy under the control of the TEF promoter is one of the excellent strategies for the high production of ε-PL. The spore germination assay demonstrated that both the short (DP = 8–20) and long size ε-PLs (DP = 26–33) effectively inhibited the spore germination of the grass pathogen *D. erythrospila* at 10 μg/mL and the oomycete *P. infestans* and the polyxenous plant pathogen *B. cinerea* at 1 μg/mL (Figure 5). To elucidate the target of -PL in the spore germination inhibition, a fluorescent staining of the germinating tubes of *D. erythrospila* with the polysaccharide-binding fluorophore Calcofluor White was performed (Figure 6). The result suggested that ε-PL interfered in the apical growth of the germinating hyphal tubes via reducing the polarized production of polysaccharide polymers on the cell-wall chitin. A similar staining pattern was also observed in the hyphal tips of *D. erythrospila* co-cultured with *E. festucae* [13]. Our ε-PL samples showed weak growth inhibition (MID = 300 μg/disk or higher doses) against the tested fungi, unlike bacteria that are known to be susceptible to ε-PL. This could be due to the relatively high lipid and polysaccharide contents in the fungal cell wall [37,38] and/or the biodegradation by the fungal protease [39]. In summary, our present studies demonstrated the effectiveness of the gene-overexpression strategy, not only for an efficient supply of the safe and potential antifungal, antibacterial, and antioomycete agent ε-PL, but also for breeding pest-tolerant pastures by infecting with genetically modified endophytes like *Epichloë*.

## 4. Materials and Methods 

### 4.1. Strains and Culture Conditions

*E. festucae* strain E437 isolated from the soft fescue *Festuca pulchella* was provided by Prof. Christopher L. Schardl (University of Kentucky, USA). *E. festucae* strain Fl1 isolated from the soft fescue *F. trachyphylla* was provided by Prof. Barry Scott (Massey University, New Zealand). For liquid culture, a mycelial block (1 × 1 cm) of *E. festucae* grown on a PDA medium (BD Difco, NJ, USA) at 23 °C was finely chopped and then inoculated in 50 mL of PD medium (BD Difco) in a 100-mL Erlenmeyer flask and incubated at 23 °C and kept on an orbital shaker at 100 rpm for 5-10 days after endophyte inoculation. Fungal pathogen *D. erythrospila* (MAFF No. 305378) was obtained from the National Institute of Agrobiological Sciences (NIAS, Japan); *Colletotrichum orbiculare* 104-T was provided by Dr. Yasuyuki Kubo (Kyoto Prefectural University, Kyoto, Japan); *Fusarium oxysporum* f. sp. *lycopersici* CK3-1, *B. cinerea* NBc1, and *Alternaria alternata* M-71 were provided by Dr. Takashi Tsuge (Chubu University, Kasugai, Japan); *Magnaporthe oryzae* Ken53-35 was from Dr. Yukio Tosa (Kobe University, Kobe, Japan); and *Aspergillus niger* AJ117065 was provided by Ajinomoto Co. (Kawasaki, Japan). All fungi were grown on PDA medium (0.4% potato extract, 2% glucose, and 1.5% agar) at 25 °C and kept in 20% glycerol at −80 °C for long storage. Oomycete pathogen *P. infestans* 08YD1 was provided by Ms. Kayo Shirai (Hokkaido Central Agricultural Experiment Station, Japan) and grown on rye media at 20 °C. *P. capsici* NBRC 30,696 was purchased from the Biological Resource Centre, National Institute of Technology and Evaluation (NBRC, Chiba, Japan) and cultured on 5% V8 vegetable juice-1.5% agar medium at 25 °C. 

### 4.2. Spectroscopic Analyses

NMR spectra were recorded on an Avance ARX400 (400 MHz for ^1^H) (Bruker Biospin, Yokohama, Japan). The chemical shifts (δ, ppm) were referenced to the internal standard (0.01% MeOH in D_2_O) at δ_H_ 3.334 and δ_C_ 49.5. MALDI-TOF MS was measured by a TOF/TOF 5800 spectrometer (AB Sciex, Foster, CA, USA) with α-cyano-4-hydroxycinnamic acid (Shimadzu Co., Tokyo, Japan) as a matrix. The calibration of MALDI-TOF MS was performed by using a mixture of PEG2000 and PEG4000 as an external calibrant. LC/MS was recorded on an HCTplus-ESI ion trap mass spectrometer (Bruker Daltonics Inc., Billerica, MA, USA) in the negative ion mode. 

### 4.3. Vector Construction and Transformation of E. festucae for Overexpression of Epls Gene

Fungal genomic DNA was isolated from mycelia grown in PD medium, as previously described [40]. PCR amplifications of genomic DNA were performed using PrimeSTAR Max DNA polymerase (Takara-Bio, Kusatsu, Japan). Plasmid pNPP195, for the constitutive expression of the *epls* gene, was prepared by cloning a 3.9-kb PCR fragment of *epls* gene amplified with primers pPN94-EfPLS-F AACCTCTAGAGGATCATGAGTCAACCTCACTCCAA and pPN94-EfPLS-R ACGTTAAGTGCGGCCTTACGCTGGAGAGGGAGACT into the pPN94 [41] digested with *Bam*HI/*Not*I using In-Fusion Cloning (Takara-Bio). The sequence of the *epls* gene is deposited in the GenBank database under the accession number LC517046. Protoplasts of *E. festucae* strains E437 and Fl1 were prepared as described previously [42] and transformed with 5 µg of the plasmid using the method previously described [43]. Transformants were selected on PDA containing 150 µg/mL hygromycin to obtain P*tef::Epls* (E437) and P*tef::Epls* (Fl1). The *vibA* gene-overexpressing transformants P*tef::VibA* (E437) and P*tef::VibA* (Fl1) were produced as previously reported [14].

### 4.4. Purification and Structure Determination of ε-PL 

Liquid cultures (400 mL in total) of the P*tef::VibA* (E437) transformant were filtered by a filter membrane unit (Millex-HA filter unit, 45 μm pore diameter; Millipore, Billerica, MA, USA), and the combined filtrates were adjusted to pH 8.5 with 6 M NaOH and adsorbed on the weakly acidic cation exchange resin Amberlite IRC-76 (Organo Corp., Tokyo, Japan) (H form, 70 mL) in a column. The resin was washed with water (420 mL) and eluted with 420 mL of 0.2 M acetic acid and then each 100 mL of 0.05, 0.1, 0.15, and 0.2 M HCl at the rate of 400 mL/h. The fractions eluted with 0.05, 0.1, and 0.15 M HCl (300 mL in total) were combined, adjusted to pH 6.5 with 1M NaOH, and concentrated to approximately 30 mL, which was dialyzed with an MWCO >1 kD membrane (Spectra/Por 7 Dialysis Membrane; Spectrum laboratories Inc., Rancho Dominquez, CA, USA). The inner solution was freeze-dried to give a brown powder (362 mg), which was subjected to reversed-phase HPLC using a Develosil ODS-HG-5 column (i.d. 20 × 250 mm) (Nomura Chemical, Seto, Aichi, Japan) eluted with 5%-30%-100%-100% MeCN (0-50-55-60 min)-0.1% triflouroacetic acid (TFA) at the flow rate of 6 mL/min and detected at 210 nm. The fractions eluted at 23–55 min were combined, concentrated, and freeze-dried to give a yellowish powder (29.09 mg), which was purified by HPLC under HILIC conditions (TSK gel Amide-80 column (i.d. 22.5 × 300 mm; Tosoh Corp., Tokyo, Japan) and 80–0% MeCN (80 min)-0.1% TFA, 6 mL/min, detected at 254 nm) to obtain ε-PL TFA salt (15.4 mg, *t*_R_ = 40.5 min) as a yellowish powder. The ε-PL samples were also purified from other transformants: P*tef::VibA* (Fl1), P*tef::Epls* (E437), and P*tef::Epls* (Fl1), by the same method. 

### 4.5. MALDI-TOF MS Analysis of ε-PL 

A portion (0.1 mg) of ε-PL TFA salt in water (100 μL) was applied on a column of strong anion exchange resin Dowex-1-X2 (0.1 mL; Dow Chemical Co., Midland, MI, USA). The column was eluted with water (100 μL × 10 fractions), and the fractions 3–7 were used for MALDI-TOF MS analysis to determine the degree of polymerization of ε-PL. The theoretical *m/z* value for each degree of polymerization was calculated as the centroid value of the constituent isotope peaks. A standard ε-PL (free polyamine form) originated from *Streptomyces albulus*, gifted by the Yokohama Research Center, JNC Co. (Yokohama, Japan), was also analyzed for comparison. 

### 4.6. Absolute Configuration

The absolute configuration of the lysine unit of ε-PL was determined by the advanced Marfey’s method [18]. A portion (0.1 mg) of ε-PL TFA salt was hydrolyzed with 6 N HCl (300 μL) at 110 °C for 2 h in a sealed tube. The reaction mixture was concentrated to dryness by nitrogen flash, and the residue was dissolved in 150 μL of water. To a portion (50 μL) were added 40 μL of 1 M NaHCO_3_ and 50 μL of a 1% solution of 1-fluoro-2,4-dinitrophenyl-5-l or D-leucinamide (L- or D-FDLA) in acetone, and the mixture was incubated at 37 °C for 1 h. The resulting mixture was treated with 20 μL of 1 M HCl and diluted with 340 μL of acetonitrile. A portion (5 μL) was applied to LC/MS analysis (Cadenza CD-C18 column (i.d. 2 × 75 mm) (Imtakt, Kyoto, Japan), 30–100% (30 min) MeCN-0.1% formic acid, and flow rate 0.15 mL/min). L-Lysine-L-DLA and L-lysine-D-DLA derivatives were eluted at 30.8 and 32.6 min, respectively. 

### 4.7. Spore Germination Assay 

Conidial suspension of *D. erythrospila* was prepared as described previously [13]. Conidiation of *B. cinerea* was induced by culturing on PDA at 23 °C under near-ultraviolet light for 2 weeks, and conidia were suspended in glucose-phosphate solution (10 mM glucose and 10 mM NaH_2_PO_4_). Zoosporangia suspension of *P. infestans* was prepared as previously described [44]. Spore suspension of pathogens (approx. 1 × 10^3^ spores/mL) are mixed with ε-PL TFA salt at an indicated concentration on a sterile concave microscope slide. The slide was placed in a humidified plastic petri dish and incubated at 23 °C for 20 h. The germination of pathogen spores was observed under a microscope BX51 (Olympus, Tokyo, Japan), and the length of germinated hyphae was analyzed by using an open-source software Image J (ver 1.50i). 

### 4.8. Cell Wall Staining 

The spores of *D. erythrospila* were treated with 50 μg/mL of ε-PL TFA salt under the above-mentioned conditions. A solution of Calcofluor White stain (0.1 mg/mL final concentration; Fluka, Taufkirchen, Germany) was applied to the spore suspension, and stained hyphae were observed under a confocal laser scanning microscopy FV1000-D (Olympus) with excitation at 405 nm and fluorescence emission between 425 and 475 nm. 

### 4.9. Antifungal Assay 

The fungi (*D. erythrospila, C. orbiculare, F. oxysporum, B. cinerea, M. oryzae*, and *A. alternata*) were cultured on PDA medium (0.4% potato extract, 2% glucose, and 1.5% agar) in a 9-cm petri dish at 25 °C for 1–12 days until colonies grew to approximately 3–4 cm in diameter. *P. capsici* NBRC 30,696 was cultured on 5% V8 vegetable juice-1.5% agar medium at 25 °C for 2 d. Paper disks (6 mm in diameter) soaked with 10 μL of a compound solution in 50% DMSO-water were placed 1 cm away from the colony front, and then they were incubated for an additional period until the colony front reached the control disk. The inhibition zone (distance between the paper disk edge and the colony front, mm) was measured to evaluate the activity. The minimum inhibitory dose (MID; μg/disk) was defined as the minimum dose that induced a weak but obvious inhibition zone (1–1.5 mm). For the black mold *A. niger*, a loopful conidia was cultured at 25 °C for 4 d on PDA medium. The colony surface was gently washed with 5 mL of 0.1% Tween 20, and a portion (0.5 mL, 5 × 10^6^ conidia) was fused in 100 mL of PDA (0.4% potato extract, 2% glucose, and 1.5% agar) at 43 °C to prepare conidia-containing agar plates. Paper disks soaked with a compound solution in 50% DMSO-water were placed on the agar plates and incubated for 2 h, and the diameter of the halo was measured. For the case that growth inhibition was observed (*D. erythrospila* and *P. capsici*), the incubation was extended until the colony covered the paper disk, and then the inhibition zones were observed through stereo microscope VH-Z100 (Keyence, Osaka, Japan). 

## Figures and Tables

**Table 1:** NMR data of ε-PL (400 MHz, deuterium oxide). HMBC: hetero-nuclear multiple-bond connectivity.

Position	δ_C_ (ppm), Type	δ_H_ (ppm), Mult. (*J*, Hz)	HMBC Correlations (to C)
α	53.3, CH	3.90, t (6.8)	γ, β, C=O
β	30.5, CH_2_	1.85, m	α, C=O
γ	21.7, CH_2_	1.37, quint (7.3)	β, ε ε
δ	28.0, CH_2_	1.55, quint (7.3)	β, ε
ε	39.1, CH_2_	3.24, m	C=O
C=O	169.6, C	-	

**Table 2:** Production and length of ε-PL produced by wild type and genetically modified *E. festucae* strains.

Strains	Production (mg/L) *^a^*	Chain length, DP *^b^*
E437 (wild type)	10.4	28–34 (32)
P*tef::VibA* (E437)	38.6	28–34 (32)
P*tef::Epls* (E437)	69.9	8–25 (15) and 26–33 (31)
Fl1 (wild type)	trace	-
P*tef::VibA* (Fl1)	2.7	8–18 (12)
P*tef*::*Epls* (Fl1)	13.9	8–20 (12)

*^a^* The amounts are as triflouroacetic acid (TFA) salts after HILIC HPLC purification; *^b^* The degree of polymerization (DP) was expressed for the ion peaks higher than 10% of the parent ion peak in MALDI-TOF MS (Figure 1). The DP for the highest ion peak (DP of largest population) within the lower or higher DP product is indicated in parenthesis.

## Supplementary Materials

The following are available online: Table S1: Antifungal activity of ε-PL. Figure S1: ^1^H-NMR spectrum of ε-PL from P*tef::VibA* (E437) (400 Hz, D_2_O). Figure S2: ^13^C-NMR spectrum of ε-PL from P*tef::VibA* (E437) (100 Hz, D_2_O). Figure S3: DQF-COSY spectrum of ε-PL from P*tef::VibA* (E437) (400 Hz, D_2_O). Figure S4: HSQC spectrum of ε-PL from P*tef::VibA* (E437) (400 MHz, D_2_O). Figure S5: HMBC spectrum of ε-PL from P*tef::VibA* (E437) (400 Hz, D_2_O). Figure S6: ^1^H-NMR spectrum of standard ε-PL (TFA salt) (400 Hz, D_2_O). Figure S7: Determination of absolute configuration of ε-PL by the advanced Marfey’s method. Figure S8: Alignment of the deduced amino acid sequences of the adenylation domain of *E. festucae* ε-PL synthetase (EfEpls) and first adenylation domain of *Bacillus licheniformis* bacitracin synthetase 2. Figure S9: Fungal growth inhibition by ε-PL against *Drechslera erythrospila* and *Phytophthora capsica*.
