# Supplementary material for: Identification of ε-Poly-L-lysine as an Antimicrobial Product from an Epichloë Endophyte and Isolation of Fungal ε-PL Synthetase Gene
Source: Molecules. 2020 Feb 25;25(5):1032. doi: 10.3390/molecules25051032 (PMC7179176; doi:10.3390/molecules25051032)
Supplement: Supplementary file 1 [file molecules-25-01032-s001.pdf]

Supplementary materials

Identification of ε-Poly-L-lysine as an Antimicrobial Product from an Epichloë Endophyte and Isolation of Fungal ε-PL Synthetase Gene

Enkhee Purev, Tatsuhiko Kondo, Daigo Takemoto, Jennifer T. Niones, Makoto Ojika*

Contents

Table S1. Antifungal activity of ε-PL

Figure S1. ^{1}H-NMR spectrum of ε-PL from P*tef::VibA* (E437) (400 Hz, D_2O)

Figure S2. ^{13}C-NMR spectrum of ε-PL from P*tef::VibA* (E437) (100 Hz, D_2O)

Figure S3. DQF-COSY spectrum of ε-PL from P*tef::VibA* (E437) (400 Hz, D_2O)

Figure S4. HSQC spectrum of ε-PL *from* P*tef::VibA* (E437) (400 MHz, D_2O)

Figure S5. HMBC spectrum of ε-PL *from* P*tef::VibA* (E437) (400 Hz, D2O)

Figure S6. ^{1}H-NMR spectrum of standard ε-PL (TFA salt) (400 Hz, D_2O)

Figure S7. Determination of absolute configuration of ε-PL by the advanced Marfey's method

Figure S8. Alignment of the deduced amino acid sequences of adenylation domain of *E. festucae* ε-PL synthetase (EfEpls) and first adenylation domain of *Bacillus licheniformis* bacitracin synthetase 2 (BlBacB, accession No. AAC06347)

Figure S9. Fungal growth inhibition by ε-PL against *Drechslera erythrospila* and *Phytophthora capsici*.

Table S1| Antifungal activity of ε-PL[a]

Fungi	MID (μg/disk)		
	**Standard ε-PL (DP = 25−35)**	**ε-PL (DP = 8−25 and 26−34)[b]**	**ε-PL (DP = 8−23)[c]**
D. erythrospila	300	300	300
P. capsici	300	300	300
C. orbiculare	>300	>300	>300
F. oxysporum	>300	>300	>300
B. cinerea	>100	>100	>100
M. oryzae	>100	>100	>100
A. alternata	>100	>100	>100
A. niger	>100	>100	>100

[a] *Expressed as minimum inhibitory dose (MID) that induces a definite inhibitory zone between paper disk and pathogen colony.*

[b] *Isolated from Ptef::Epls (E437) transformant. Degree of polymerization (DP) is indicated in parenthesis.*

[c] *Isolated from Ptef::Epls (Fl1) transformant.*

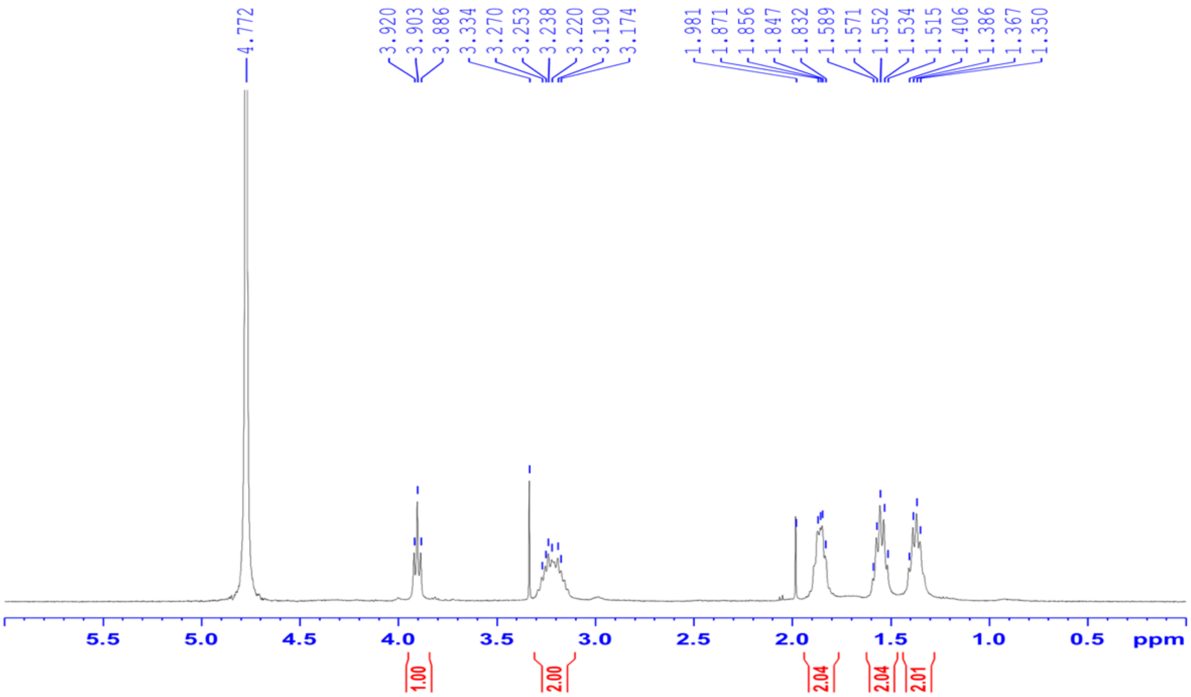

Figure S1| ^{1}H-NMR spectrum of ε-PL from P*tef::VibA* (E437) (400 Hz, D_2O)

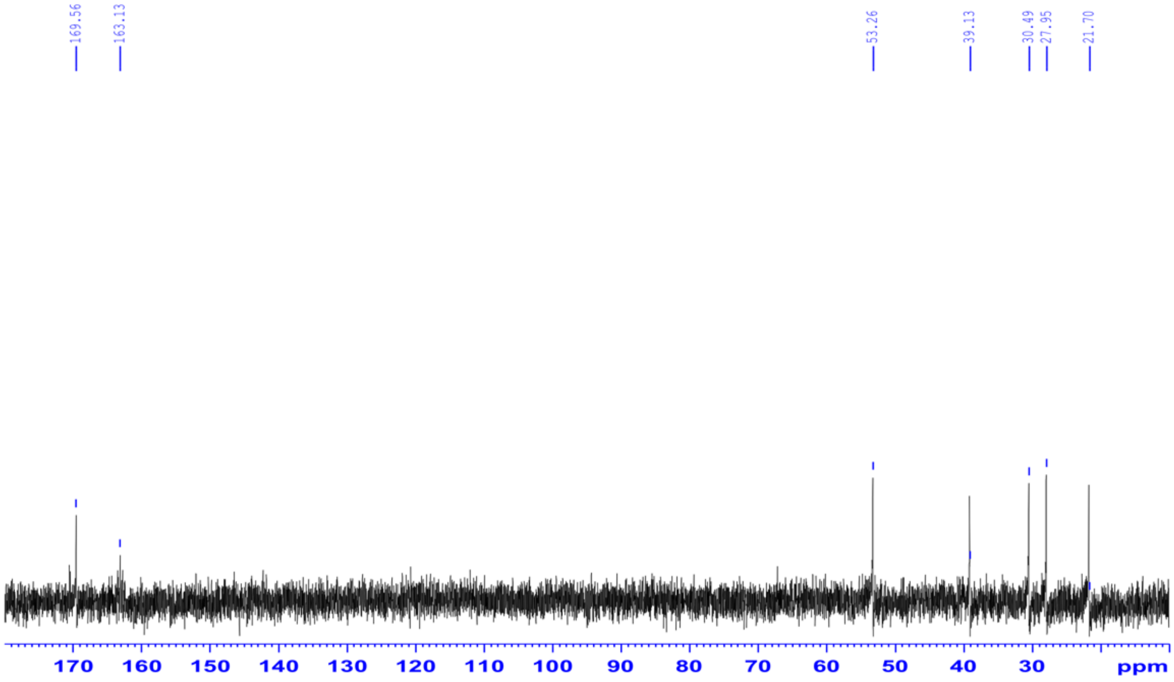

Figure S2| ^{13}C-NMR spectrum of ε-PL from P*tef::VibA* (E437) (100 Hz, D_2O)

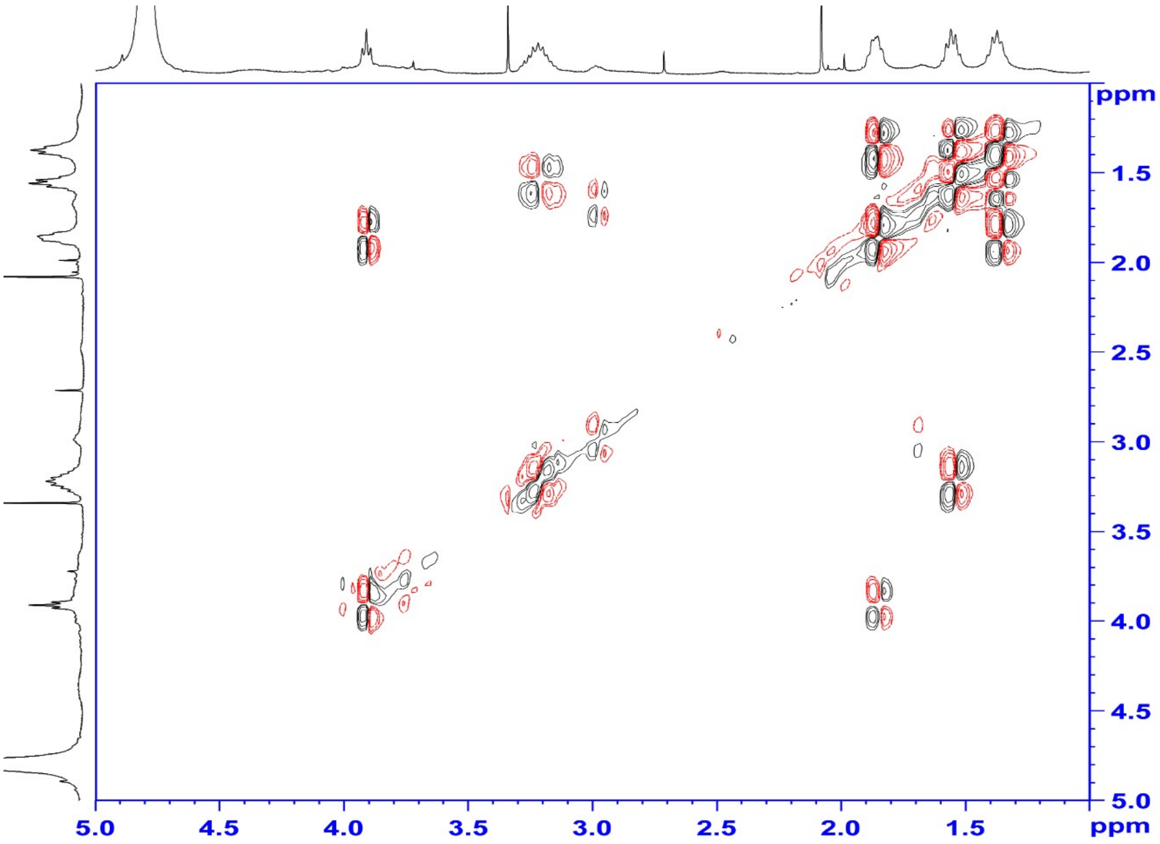

Figure S3| DQF-COSY spectrum of ε-PL from P*tef::VibA* (E437) (400 Hz, D_2O)

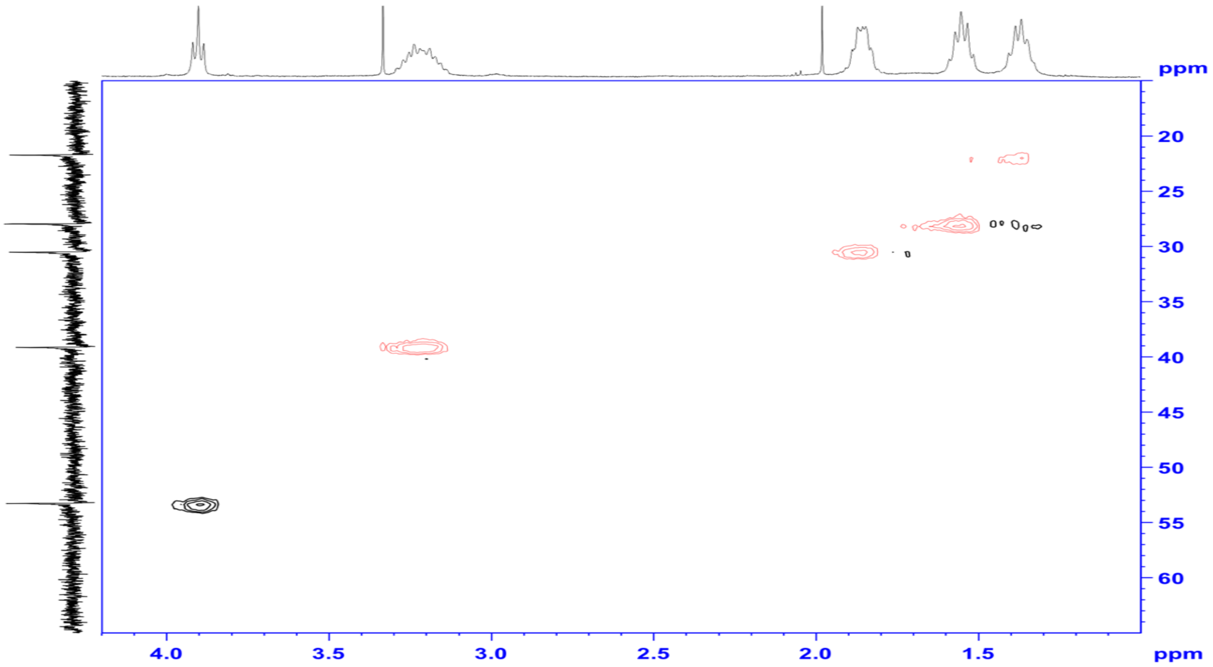

Figure S4| HSQC spectrum of ε-PL from P*tef::VibA* (E437) (400 MHz, D_2O)

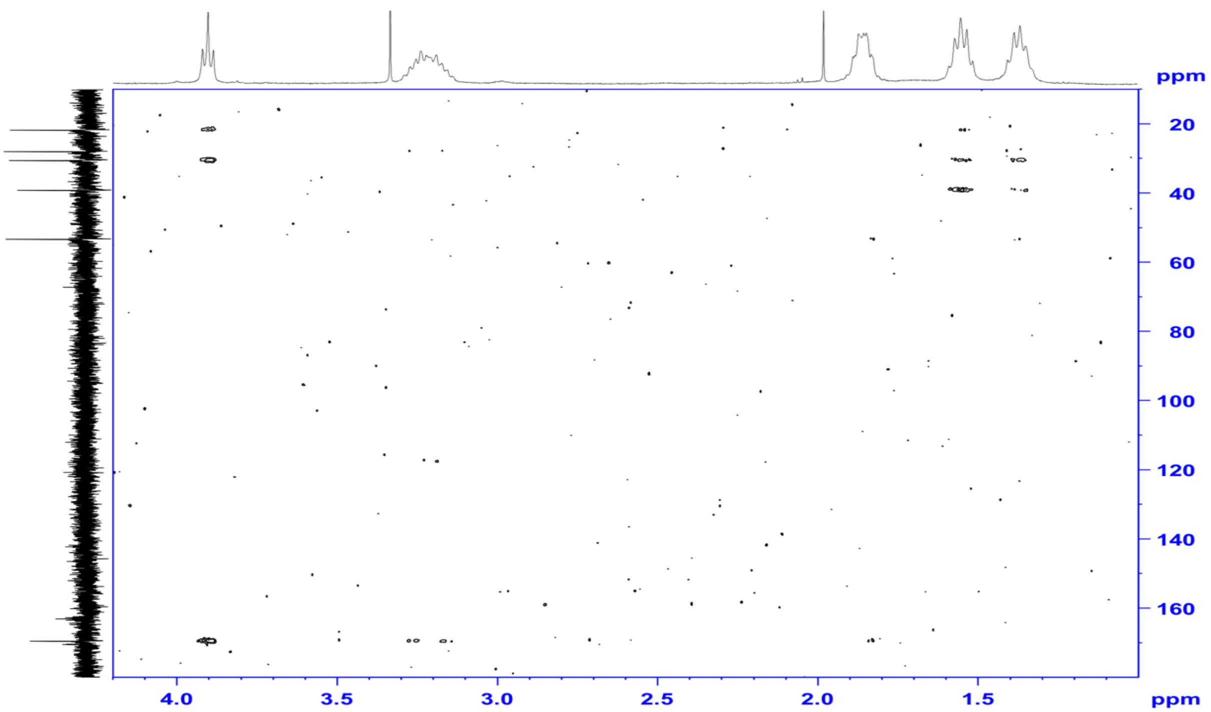

Figure S51| HMBC spectrum of ε-PL from P*tef::VibA* (E437) (400 Hz, D_2O)

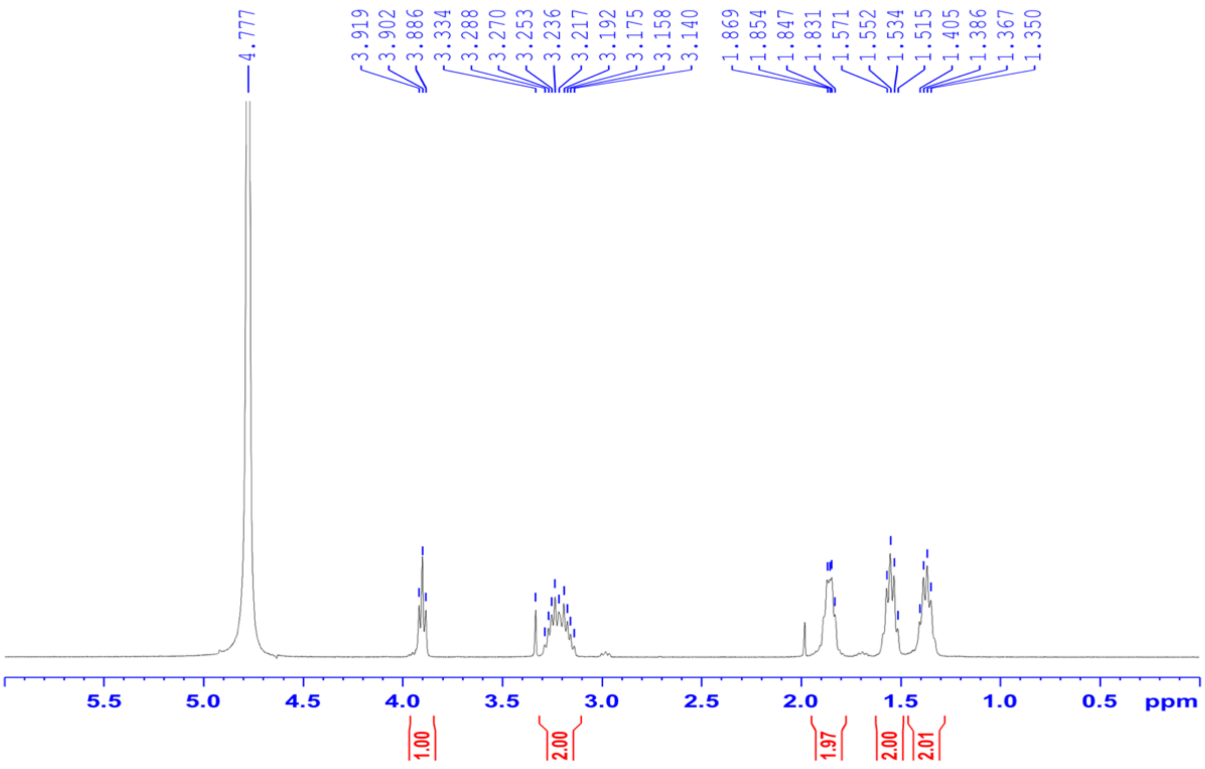

Figure S6| ^{1}H-NMR spectrum of standard ε-PL from (400 Hz, D_2O)

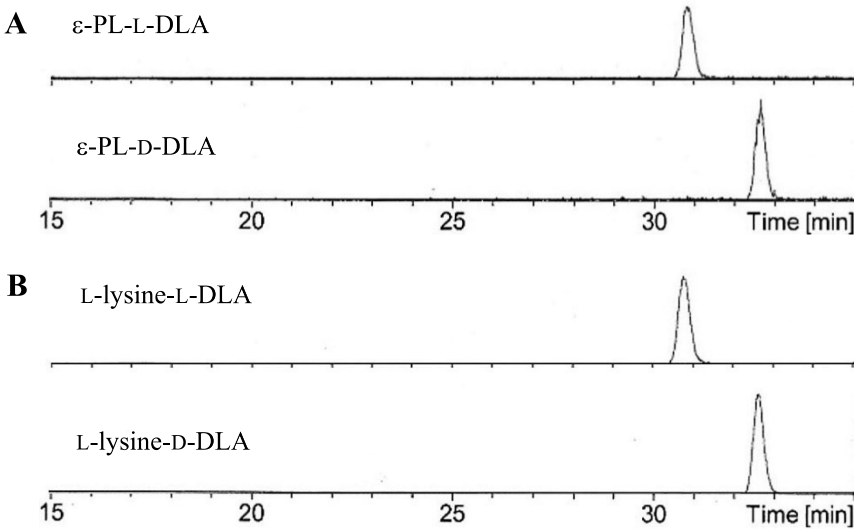

Figure S7| Determination of absolute configuration of ε-PL by the advanced Marfey's method. Extracted ion chromatograms obtained by LC/MS analysis of L- and D-DLA derivatives of acid hydrolysate of ε-PL from P*tef::VibA* (E437) (**A**) and L-lysine (**B**).

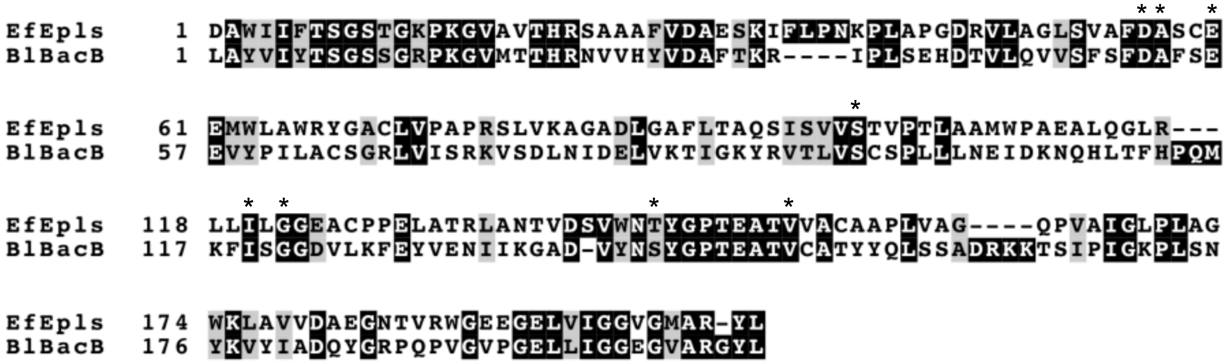

Figure S8| Alignment of the deduced amino acid sequences of adenylation domain of *E. festucae* ε-PL synthetase (EfEpls) and the first adenylation domain of *Bacillus licheniformis* bacitracin synthetase 2 (BlBacB, accession No. AAC06347). Amino acid residues for the lysine-binding pocket in the first A domain of BlBacB are indicated by asterisks.

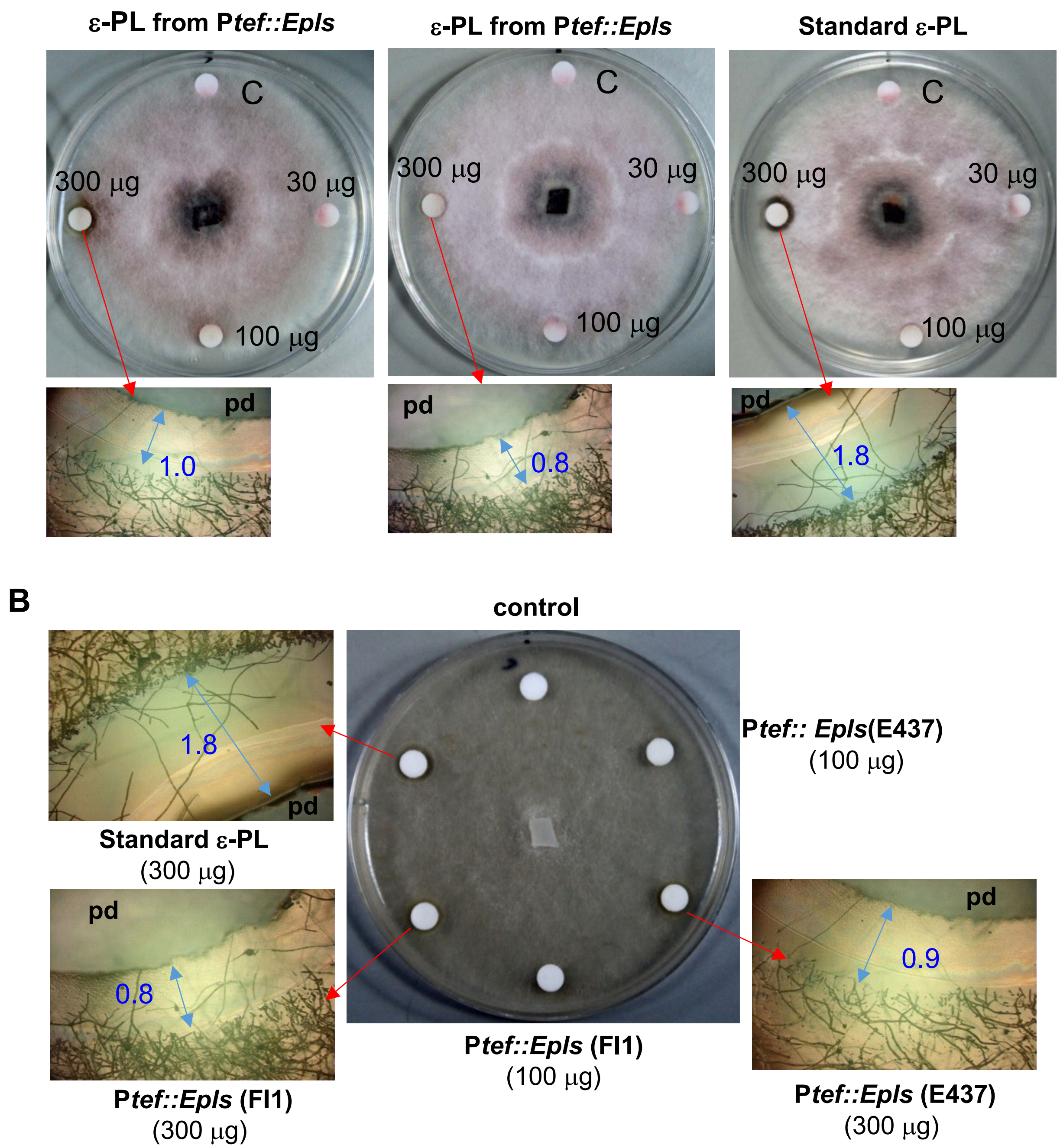

Figure S9| Fungal growth inhibition by ε-PL against **(A)** *Drechslera erythrospila* and **(B)** *Phytophthora capsici*. Effects of the ε-PL was observed 3 (**A**) or 5 days (**B**) after applying sample-containing disks. Inhibition zones between paper disk edge and colony front (in mm) around the paper disk ("pd") were observed through a stereo microscope.
